# Big Data Health Care Innovations: Performance Dashboarding as a Process of Collective Sensemaking

**DOI:** 10.2196/30201

**Published:** 2022-02-22

**Authors:** Hilco J van Elten, Sandra Sülz, Erik M van Raaij, Rik Wehrens

**Affiliations:** 1 Erasmus School of Health Policy & Management Erasmus University Rotterdam Netherlands; 2 Rotterdam School of Management Erasmus University Rotterdam Netherlands

**Keywords:** dashboarding, big data, balanced scorecard, performance measurement, key performance indicators, digital health, dashboards, knowledge translation, health information, stakeholders, health care

## Abstract

Big data is poised to revolutionize health care, and performance dashboards can be an important tool to manage big data innovations. Dashboards show the progress being made and provide critical management information about effectiveness and efficiency. However, performance dashboards are more than just a clear and straightforward representation of performance in the health care context. Instead, the development and maintenance of informative dashboards can be more productively viewed as an interactive and iterative process involving all stakeholders. We refer to this process as *dashboarding* and reflect on our learnings within a large European Union–funded project. Within this project, multiple big data applications in health care are being developed, piloted, and scaled up. In this paper, we discuss the ways in which we cope with the inherent sensitivities and tensions surrounding dashboarding in such a dynamic environment.

**International Registered Report Identifier (IRRID):**

RR2-10.2196/16779

## Introduction

Big data innovations in health care are on the rise, with benefits that include providing clinical intelligence about a patient’s risk of future adverse health events [1], empowering patients through big data–driven eHealth applications [2], increasing interest in clinical prediction tools [3], and reducing costs by leveraging big data to detect fraud, abuse, waste, and errors in health insurance claims [4]. Recently, China has used big data technology in its attempts to prevent and control the spread of COVID-19 [5]. All in all, big data innovations in health care promise to address the rising demand for high-quality health care services and to reduce the accompanying costs of care. This makes big data appealing for governments and funding agencies, who are increasingly investing in large-scale, interdisciplinary projects and consortia that seek to develop big data innovations, implement them in the health care field, and ideally demonstrate their desired impact. Such projects usually involve a multitude of stakeholders who, in addition to the project goal, have their own objectives, such as creating social or economic impact, publishing research, or commercializing the innovation. These large investments and diverse goals and objectives call for close monitoring of the overall progress and performance of projects. Such performance monitoring is frequently done utilizing dashboards containing key performance indicators (KPIs).

Dashboards provide a visual overview of the information needed for performance management, and by doing so, facilitate decision-making and project management. In the health care field, the use of dashboards is manifold. Types of dashboard include unit-specific nursing performance dashboards [6], hospital-wide or disease-specific quality and safety diagnostics dashboards [7,8], and population-level maternal and newborn dashboards [9]. Management literature has emphasized the use of performance dashboards to provide an overview of relevant KPIs and to enable performance management in hospitals [7,10,11]. As such, dashboards are often preferred as managerial instruments to gain insights about progress and performance.

Despite their appeal and aura of rationality, performance dashboards are not simply a clear and straightforward representation of health care performance. Indeed, this point has gained traction within recent accounting literature [12]. Building on the insights from accounting literature, critical management studies, and science and technology studies, we argue that one cannot simply design a dashboard to capture the productivity or efficiency of something as inherently complex and multifaceted as the performance of big data innovations in health care [12]. At the same time, dashboards continue to have enormous appeal, both for funders of research and implementation projects (who perceive them as providing tangible outcomes to account for investments made) and for health care managers (who view them as instruments that can provide valuable information). Therefore, such tools cannot simply be dismissed because of the inherent challenges mentioned above.

In this paper, we introduce our perspective on the development of systems for monitoring big data innovation projects in health care through performance dashboards and discuss caveats concerning these systems. Our perspective focuses on the social dimension of this interactive process, that is, we do not delve into the technical dimensions, such as techniques for data analytics and visualization [13]. Importantly, we view the performance dashboards as the *means* to monitor big data innovation projects. In our paper, they are not the *results* of big data innovations.

We argue that the development of such dashboards does not match the prevalent rationalist narrative usually assumed. Far from being a series of rational decisions, this development can be better understood as a process of collective sensemaking. While the idea of sensemaking has strong roots in organizational psychology, in which the notion is used to study the microfoundations of organizing, we use the notion here to point toward a more general focus on interpretive forms of research that address how meanings are negotiated and constructed [14]. Nevertheless, although we do not build on the full theoretical framework associated with sensemaking, our use also shares affinities with organizational research that describes sensemaking as being “not about truth and getting it right,” but instead “about continued redrafting of an emerging story so that it becomes more comprehensive, incorporates more of the observed data, and is more resilient in the face of criticism” [15]. Therefore, we suggest a more pragmatic approach toward the development and use of performance dashboards. In this process, for which we propose the term *dashboarding*, many decisions need to be made jointly with the most important stakeholders. Anything but a linear sequence of decisions, this is a necessarily iterative, recursive process of moving back and forth to find out which indicators are feasible, acceptable, measurable, and informative.

Our viewpoint is based on both our familiarity with the literature regarding the goals, drivers, and problematic aspects of using dashboards as instruments for performance measurement, and on our experiences as researchers and dashboard developers in a large-scale, 3-year European project aimed at developing big data applications in health care, titled “Big Data for Medical Analytics,” shortened to “BigMedilytics” (this project was approved by the ethics board of the Erasmus Medical Centre [MEC-2018-056] and the ethics review board of Erasmus University [EA18-01]). Drawing on our experiences in the development, tailoring, and further modification of performance dashboards for 12 pilot projects to develop big data innovations in health care in 8 European countries, our aim is to increase awareness of tensions and to develop sensitivities among academics, clinicians, and practitioners involved in performance measurement of health care innovations, especially those involving big data. We draw on the numerous discussions we have had with the research team and pilot partners, our experiences in organizing various pilot project–specific workshops discussing dashboard designs at various stages, and the many emails, phone calls, and various other interactions we have had with pilot project teams over the last years.

## The BigMedilytics Project

Our empirical setting is the 3-year BigMedilytics project, funded by the European Union. This project aims to “enhance patient outcomes and increase productivity in the health sector by applying big data technologies to complex datasets while ensuring security and privacy of personal data” [16]. The entire consortium consists of 35 different entities, ranging from health care providers, technology companies, and insurers to research institutes and universities. At the core of the consortium are 12 pilot projects that develop big data innovations in health care in 8 European countries, divided into 3 areas: population health and chronic disease management, oncology, and health care services industrialization, as described in the underlying protocol paper [13]. The big data technologies include systems to derive predictive models, clinical decision support systems, and real-time asset location systems.

### The Inherent Complexities of Dashboarding

While performance dashboards can provide useful insights into the effectiveness and quality improvement potential of big data innovations, rational, straightforward design and implementation of these dashboards is problematic for several reasons. Building on literature in accounting, critical management studies and science and technology studies, we identify 4 important insights that deeply problematize the idea that dashboards simply “capture” performance. First, performance is an inherently debated and complex concept [17,18]. At the core of health care systems are health care organizations that seek to fulfill multiple, sometimes even conflicting aims. As such, tensions are likely to arise between what counts as “performance” in different contexts. This makes performance monitoring and performance management inherently complex. Second, there is always a trade-off between validity and feasibility. The search for exhaustive validation of a dashboard comes at the cost of practical feasibility; when measurement aims to provide a flawless map of the organization’s landscape, this often hinders practical utility and enhances strategic behavior [19,20]. Third, there is some tension between using performance dashboards internally for learning purposes and using dashboards externally as bases for monitoring and accountability [21]. In order to learn from performance dashboards, it is vital to stimulate discussions based on the performance scores. Using performance measurement as an accountability tool is an “external” use of the dashboard, in which those who monitor the project use the dashboard to steer and direct. Using performance measurement as an accountability and control tool is likely to corrode and corrupt the performance indicators, undermining the conditions required for quality improvement [21]. Fourth, performance monitoring is never simply a representation of reality, but also shapes and structures the reality to be acted upon in a process of coconstitution [22,23]. Dashboards never only represent performance or particular aspects of performance; they are also social facts that generate actions and reactions, for example, by defining managerial priorities and by reconfiguring work routines and relationships between actors [24,25]. They are thus “performative” [24,25] in the sense that they do not only represent aspects of organizational performance but also help shape and define the very aspects of performance that come to matter—an aspect that is also recognized within the accounting literature [26].

### Three Persistent Tensions

Based on our experiences in the BigMedilytics project, we will describe 3 persistent tensions that we and other actors in the consortium experienced while working toward performance dashboards that met the dual requirements of being useful to pilot partners while allowing for some form of assessment regarding goals and achievements. After this description, we will outline the main implications, reiterate our suggestion that a more pragmatic perspective on dashboarding should be developed, and discuss the implications of this perspective for academics, clinicians, and practitioners involved in the performance measurement of health care innovations.

#### Tension 1: Navigating Between Divergent Stakeholder Views and Expectations

The consortium that we were a part of included the developers of a range of big data innovation projects, which involved, like many similar projects, various stakeholder groups from academia, government, and industry. At the core of the consortium were 12 big data pilot projects to develop and implement big data innovations in 8 European countries: Austria, France, Germany, Ireland, the Netherlands, Spain, Sweden, and the United Kingdom. These pilot projects were divided into 3 themes: population health and chronic disease management (n=5), oncology (n=3), and health care services industrialization (n=4). Each of the 12 pilot project teams included several members, introducing a multitude of experiences and expertise.

Consortia necessarily bring together a range of stakeholders, who each have their own specific ideas about the goals of the project. Many innovation projects, not just those organized as consortia, involve internal or external funding. In order to apply for funding, stakeholders need to align goals with each other. An inherent feature of the application and alignment process is time constraint, which hampers the ability to acquire all the information needed in order to make a fully informed decision. Consequently, one must settle on a goal that is deemed acceptable for all groups given the information available at the time. This necessitates broadly defined goals that require further specification over time. In our case, the BigMedilytics consortium aligned on the broad and ambitious aim to improve health care in Europe through big data and “demonstrate an increase in healthcare productivity between 20% and 63%” [27].

But as time unfolded, it became evident that people attributed different meanings to the term *productivity*, which affected the way they conceptualized and operationalized it. One prominent definition of productivity considers it to be the ratio of input to output and to thereby express how efficiently resources are used [28,29]. Defining the overall project goal in such strong economic and engineering language, however, did not match the perception of how individual pilot projects could contribute to achieving productivity gains by deploying big data. With the big data innovations organized in 12 very different pilot projects, various idiosyncratic features needed to somehow be reflected in how the productivity of the projects was measured. A one-size-fits-all approach was considered inappropriate and a certain degree of divergence was deemed inevitable. One might raise the argument that this divergence was a consequence of the heterogeneity of the pilot projects, and consider that homogeneous pilot projects would not face this tension. While this argument has some appeal, we know from the literature that even in similar settings, different opinions prevail on what constitutes productivity [30], performance [31], and quality of care [32]. Consequently, some divergence is inevitable if one is to maintain stakeholders’ support and engagement. Stakeholders need to see their pilot project–specific contribution to the overall goal adequately reflected.

This divergence needs to be managed. The more pilot project–specific idiosyncrasies are considered, the better pilot project–specific developments can be monitored and the more precisely we can formulate a pilot project–specific conclusion. By the same token, the more pilot project–specific idiosyncrasies are considered, and the more pilot projects diverge, the more challenging it becomes to converge again and draw conclusions across pilot projects. This indicates a tension: if divergence amongst pilot projects is too high, goal congruence might be compromised. On the other hand, not allowing any divergence would amount to the one-size-fits-all approach that was already considered inappropriate. Managing this tension thus calls for a compromise that allows for some divergence but at the same time enables alignment between stakeholders and convergence toward the overall project goal.

Striving for convergence but simultaneously allowing for necessary divergence resulted in the following approach: we applied a broader angle and considered performance along multiple dimensions. While the multi-dimensionality of the performance framework thus increased the likelihood that stakeholders considered the framework acceptable, it initiated a discussion on what type of performance dimension adequately captured the objectives and intentions of the big data innovations. This called for a compromise: we decided to align on dimensions broad enough that they applied to multiple pilot projects (in step 1), but also recognized that we needed to give pilot projects the possibility to define KPIs within these dimensions that adequately captured their unique qualities (in step 2). We used a balanced scorecard (BSC) [33] to monitor multiple performance dimensions and tailored its design by determining pilot project–specific KPIs. The BSC [33,34] is a widely applied framework, primarily in for-profit business organizations, and consists of a set of KPIs that gives top managers a fast but comprehensive view of the organization. The set of KPIs includes financial performance measures that show the results of actions already taken. The BSC complements the financial measures with operational, process, and quality measures of various performance dimensions that are the drivers of future financial performance.

Although popularized in for-profit business organizations, the application and study of BSCs in the health care industry is not new. In the health care industry, most prominent descriptions of BSCs refer to monitoring quality aspects of performance. For example, Chong et al [35] found that “measuring the quality of a hospital is an important but exceedingly difficult task. Different methods of capturing quality have been devised, including composite scores of compliance with various quality indicators to the adoption of BSC techniques from the business world.” Also, Fernando et al [36] support this idea by claiming that scorecards are used by institutions “for the purposes of monitoring clinical performance and driving quality improvement.”

#### Tension 2: Navigating Between Timely and Meaningful Data Collection

For each of the performance dimensions of the BSC (ie, patient satisfaction, patient outcomes, process outcomes, and financial outcomes), we determined pilot project–specific KPIs in close collaboration with the pilot project stakeholders via an iterative procedure. For example, pilot projects that related to population health, chronic disease, and oncology often included long-term financial measures (eg, the projected cost of care over 10 years) next to short-term measures, such as the average cost per patient. In most pilot projects, patients were expected to be directly impacted by the big data innovation. In these cases, patient satisfaction surveys and patient-reported measures were relevant to measure the dimension “patient satisfaction.” Mortality was a typical measure for the dimension “patient outcomes.” In contrast, the pilot projects related to industrialization did not measure KPIs for the “patient satisfaction” dimension, as patients were not directly impacted by the big data innovations, but instead focused on process outcomes (such as completion time for diagnoses).

Through this collaboration we aimed to ensure the relevance of the selected KPIs to the pilot projects, as well as increase commitment of the pilot project teams to the dashboard. This commitment was key as the pilot project teams were supposed to report on these KPIs every 6 months. The suggestion from the project consortium was to rely on standardized lists of KPIs. These KPIs were often already available in the reporting and management systems used in the pilot projects or the organizational bodies (eg, hospitals) to which the pilot projects belonged. Such availability would make reliable data collection very efficient. However, efficient data collection of readily available KPIs is not the same thing as valid and meaningful data collection of KPIs that capture the actual performance and progress of pilot projects.

Therefore, we engaged in a discussion with the pilot project teams about the strategic aims of the pilot projects to make an initial assessment of priorities and feasibility. First, we organized a series of workshops where we presented the 4 dimensions to pilot project stakeholders and let the stakeholders openly brainstorm potential KPIs for each of these dimensions. Then, we compared the KPIs across pilot projects to identify similarities and differences and inquired whether KPIs that were suggested in other pilot projects might also be relevant for the pilot project in question. As a final step in the development of the KPI dashboard, the researchers and the pilot project stakeholders agreed on a set of pilot project–specific KPIs that were deemed relevant to the pilot project in question and for which reliable data were expected to be periodically collected.

After a baseline measurement of the KPIs, which provided a crucial anchor point for benchmarking the pilot project’s subsequent performance, the KPIs were supposed to be updated periodically using the same operating procedures and definitions. Receiving periodic updates has, however, frequently been challenging. Reasons for this were manifold: updating the KPIs was not deemed meaningful at that point in time; pilot projects were partially dependent on other entities to deliver the data, which prevented timely data submission; databases were not updated as frequently as intended; information technology systems were changing over time, which required modifying the operating procedures to gather the KPI data; and some KPIs turned out to be unreliable and needed to be revised whereas other KPIs—despite all good intentions—simply could not yet be measured due to a lack of data. Consequently, the set of KPIs changed over time, altering the design of the dashboard and reducing the reliability of the measurement over time.

In contrast to the idea that KPIs can capture performance and progress over time, this project made clear that a set of KPIs—even if tailored to a specific context—cannot be considered to be static, but must rather be thought of as dynamic, evolving, and changing. This holds true specifically for big data innovations in which more and more learnings are generated as time passes and new data become available. The trade-off between validity, reliability, and feasibility becomes apparent: KPIs that have initially been considered a valid indicator of performance might be more reliable due to the amount of data available, but they may also no longer be considered to be valid because they fail to capture state-of-the-art performance. Again, this tension points toward the need to understand KPI dashboards in process terms: as a dynamic process of “dashboarding” that requires adaptations over time in order to remain informative given the state of the art.

#### Tension 3: Navigating Between Different Dashboarding Needs and Purposes

Through collaboration with each individual pilot project team, we wanted to develop dashboards that would do justice to the idiosyncratic nature of the pilot projects and would be loaded with relevant and timely datapoints for an informative performance analysis. Performance analyses can be informative for the members of each pilot project team, enabling them to learn and improve, and for those monitoring progress across pilot projects. Serving both these needs, however, turned out to be difficult. Monitoring progress across pilot projects required comparability that could only be achieved by choosing a set of KPIs that was a compromise; they did not fit individual projects perfectly, making them a suboptimal choice. Learning and improving within a pilot project, however, requires selecting KPIs that are as closely related to the innovation as possible. Since big data innovations themselves do not improve health care directly, but rather contribute to improvements by changing the information used in decision-making, the way the innovation is embedded needs to be reflected in the KPIs. For example, the BigMedilytics “asset management” pilot project deployed a track-and-trace interface that professional caregivers used to locate medical equipment. As such, the interface was directly involved in a caregiver’s search process and could thereby directly influence the health care process. There were also pilot projects, however, where such direct embedding was not the case. The purpose of the “stroke workflow” pilot project was to use big data to identify bottlenecks in the workflow. As such, big data was used as a diagnostic tool to identify problematic areas in the health care process. This identification step revealed changes in the health care process that needed to be made, yet the big data innovation itself was not directly involved in health care delivery. Similar arguments pertain to other BigMedilytics pilot projects, showing that the way in which the innovation is used and how it affects the health care process differ in specific situations.

With pilot project members closely involved in the KPI selection process, one would expect the dashboards to be used for learning and improvement purposes. However, we did not observe strong indications of this. In our experience over the years, the dashboards were more often perceived as a managerial necessity for which KPI data needed to be provided for monitoring purposes. Ironically, we also did not observe strong indications of dashboards being used to oversee progress across pilot projects, which seems to indicate that the overall purpose of the dashboard remained unclear and that its potential to facilitate learning and monitor progress was insufficiently realized.

## Implications

Based on our experience with developing performance dashboards for 12 big data innovation pilots, we have argued for the need to develop a more pragmatic, process-based perspective on performance dashboards. We describe this as the notion of “dashboarding.” This perspective recognizes that despite its rationalistic aura, developing, tailoring, and modifying performance dashboards is in essence an unpredictable, messy, and iterative process that (1) involves a wide range of stakeholders with often diverging goals and expectations, (2) calls for situation-specific assessments of the balance between efficient and meaningful data collection, and (3) comes with struggles with hard-to-reconcile demands, such as the need to monitor achievements across pilot projects to account for investments made versus the need to provide tailored insights to help specific pilot project teams evaluate and improve their performance.

What are the implications of this perspective for academics, clinicians, and practitioners involved in the performance measurement of health care innovations? The first implication is that those involved in the process of dashboarding need to develop the political sensitivity to acknowledge and manage differences in interests and objectives among various stakeholders that are directly involved in and indirectly affected by the dashboard. Importantly, this goes beyond the idea of cocreation with stakeholders. While incorporating stakeholders early on is an important condition for generating support, it is by no means a panacea [37]. With an increasing number of stakeholders involved, the diversity in interests and expectations is likely to increase as well. Obviously, this may cause tensions if interests and expectations are diverging or even conflicting. In line with related literature [7,37], we therefore suggest involving stakeholders in co-designing dashboards, but we also stress that this requires careful expectation management, sensitivity toward different needs and requirements, and persistence in navigating between different perspectives and interests. Collaborative design brings together specialists and generalists from various backgrounds who share knowledge of the design process as well as the design content in order to create shared understanding of both aspects [38]. Such principles of collaborative design can be combined with principles of participatory design, which refers to the participation of prospective users, who become true participants and not just informants in the design process [39]. We involved future dashboard users from different disciplines (including data scientists and medical professionals) to some extent but would recommend more extensive and more explicit use of collaborative and participatory design principles in the dashboarding process.

The second implication of the pragmatic perspective we propose relates to the iterative nature of dashboarding. Developing dashboards iteratively through multiple stages of refinement does not align well with the logic of “projectification” that often underlies large-scale programs like the BigMedilytics program [40,41]. The notion of projectification refers to a mode of science governance that sets the concept of the “project” as its basic organizing principle [42]. As such, it highlights not only the ubiquity of the project format, but also points to underlying instrumental reasoning and a rationalistic attitude toward predictability (eg, an emphasis on activities leading to “milestones” and “deliverables”) that is often considered to be in tension with other values of research and innovations (such as academic freedom and creativity) [40,43]. This attitude is at odds with the dynamic and continuously evolving environment organizations find themselves in. Far from a controlled, experimental setting, pilot projects exist among a swirl of other projects, initiatives, developments, and changes. In the BigMedilytics program, for instance, all pilot projects were confronted with two major changes: the introduction of the General Data Protection Regulation and the COVID-19 pandemic. The main implication of this is that researchers and dashboard developers would benefit from building in more leeway for interim changes. Thinking through the implications of this to the fullest extent would also require funders and other policy makers to think about innovative ways of funding research and technology development that do not take the logic of projectification as a pre-eminent reality.

The third implication relates to the need for flexibility in dashboarding. Dashboards are used for different purposes that range from providing external accountability and internal benchmarking to enabling improvement initiatives [7]. Dashboards are also frequently considered to be informational tools that stimulate discussion and critical reflection [6]. As such, dashboards need to be flexible enough to cater to multiple purposes and different stakeholder needs [7]. This flexibility requires a specific view on progress monitoring via performance dashboards. Instead of striving for comparability across dashboards in a volatile environment (which is particularly relevant for big data as well as health care), progress monitoring can also be achieved with a focus on continuous improvement. If each project team develops its own set of KPIs (with some steering, as described above), a dashboard will emerge that will be deemed informative by the project team. Project teams should also regularly reflect on whether the set of KPIs is still informative and whether adaptations are necessary. The reasons for incorporating a new KPI or dropping an old one reveal important insights about the progress that can even go beyond the indications derived from comparing KPI scores across project teams or time.

Future research might investigate which specific approaches, tools, techniques, programming languages, and design environments are most effective for such a collaborative, iterative and flexible dashboarding process. Successful cases of dashboard development, including details on the technologies used, are available in the literature [7,44-46], but there remains a need for a systematic comparison of dashboarding approaches.

## Conclusion

Dashboarding is a dynamic process that features various tensions. Instead of neglecting these tensions, we plead for reflection upon them and navigation through them. Our recommendations therefore do not come in the form of a magic bullet and do not offer clear-cut solutions but are rather a description of 3 sensitivities that performance dashboard designers should develop to handle the tensions involved in the process of dashboarding. Capitalizing on these sensitivities will lead to dashboarding that is iterative, integrative, and informative.

## Abbreviations

BSC: balanced scorecard
KPI: key performance indicator
